# Enhanced Eye Velocity in Head Impulse Testing—A Possible Indicator of Endolymphatic Hydrops

**DOI:** 10.3389/fsurg.2021.666390

**Published:** 2021-05-07

**Authors:** Ian S. Curthoys, Leonardo Manzari, Jorge Rey-Martinez, Julia Dlugaiczyk, Ann M. Burgess

**Affiliations:** ^1^Vestibular Research Laboratory, School of Psychology, The University of Sydney, Sydney, NSW, Australia; ^2^MSA ENT Academy Center, Cassino, Italy; ^3^Otoneurology Unit, Otolaryngology Department, Hospital Universitario Donostia, San Sebastian, Spain; ^4^Department of Otorhinolaryngology, Head and Neck Surgery, University Hospital Zurich, University of Zurich, Zürich, Switzerland

**Keywords:** endolymphatic hydrops, Menière's Disease, vHIT, semicircular canal testing, VOR, vestibular

## Abstract

**Introduction:** On video head impulse testing (vHIT) of semicircular canal function, some patients reliably show enhanced eye velocity and so VOR gains >1.0. Modeling and imaging indicate this could be due to endolymphatic hydrops. Oral glycerol reduces membranous labyrinth volume and reduces cochlear symptoms of hydrops, so we tested whether oral glycerol reduced the enhanced vHIT eye velocity.

**Study Design:** Prospective clinical study and retrospective analysis of patient data.

**Methods:** Patients with enhanced eye velocity during horizontal vHIT were enrolled (*n* = 9, 17 ears) and given orally 86% glycerol, 1.5 mL/kg of body weight, dissolved 1:1 in physiological saline. Horizontal vHIT testing was performed before glycerol intake (time 0), then at intervals of 1, 2, and 3 h after the oral glycerol intake. Control patients with enhanced eye velocity (*n* = 4, 6 ears) received water and were tested at the same intervals. To provide an objective index of enhanced eye velocity we used a measure of VOR gain which captures the enhanced eye velocity which is so clear on inspecting the eye velocity records. We call this measure the initial VOR gain and it is defined as: (the ratio of peak eye velocity to the value of head velocity at the time of peak eye velocity). The responses of other patients who showed enhanced eye velocity during routine clinical testing were analyzed to try to identify how the enhancement occurred.

**Results:** We found that oral glycerol caused, on average, a significant reduction in the enhanced eye velocity response, whereas water caused no systematic change. The enhanced eye velocity during the head impulses is due in some patients to a compensatory saccade-like response during the increasing head velocity.

**Conclusion:** The significant reduction in enhanced eye velocity during head impulse testing following oral glycerol is consistent with the hypothesis that the enhanced eye velocity in vHIT may be caused by endolymphatic hydrops.

## Introduction

An enhanced eye velocity response to stimulation of the semicircular canals by angular acceleration has been reported in patients with Menière's Disease (MD) (1–3). Evidence for enhanced eye velocity in the video head impulse test (vHIT) of horizontal semicircular canal function (vHIT) with very high angular accelerations (up to 5,000 deg/s^2^) has also been reported (4–7). The prevalence of this increased enhanced eye velocity has now been documented by a large study (7) showing a prevalence for enhanced eye velocity in vHIT testing of patients with MD. In these patients the result was not due to poor calibration or artifacts in testing, such as goggle slip (8, 9) since the testers were very experienced clinicians who carefully checked for calibration errors or goggle slip. It was not due to testing with a very close viewing distance—which acts to increase VOR gain (10, 11). Repeated vHIT tests show that enhanced eye velocity is a characteristic response pattern for individual patients, and the consistency of such a response can be seen in the repeated test results of one patient on different occasions (4). In these patients during a head impulse, the eye velocity exceeds the head velocity, resulting in a VOR gain >1.0, where the area VOR gain is defined as (the area under the eye velocity record divided by the area under the head velocity record). It appears that enhanced eye velocity in vHIT testing is rarely found in testing healthy subjects (7).

The focus of the present study was on the possible cause of such enhanced eye velocity. Our hypothesis is that it may be due to endolymphatic hydrops (ELH). Imaging of the labyrinth of two patients with enhanced eye velocity has shown ELH (4). The hypothesis is that ELH alters the hydrodynamic load on the cupula during an angular acceleration and so results in an increased eye velocity (4, 5, 12). That hypothesis is supported by fluid dynamical modeling of the effect of ELH on vHIT responses showing that ELH resulted in enhanced eye velocity responses similar to the enhanced responses actually obtained from patients with MRI-confirmed ELH (4, 5, 12). To further test this hypothesis, we sought in patients showing enhanced eye velocity to modify labyrinth volume experimentally by using oral glycerol and testing what effect that modification had on their enhanced eye velocity response.

There is anatomical evidence from guinea pig studies that glycerol acts to reduce membranous labyrinth volume (13, 14). In patients the glycerol dehydration test has been used to indicate ELH in auditory testing by measuring auditory thresholds before and after oral intake of glycerol. The reduction of thresholds for low frequencies is an indicator of probable ELH (15, 16). Objective measures show that oral glycerol caused changes of the cochlear electrophysiological indicator of ELH (the SP/AP ratio of the ECochG during glycerol) (17). There are vestibular parallels to these auditory changes after glycerol—VEMP amplitude increases (18–22). So, we reasoned that the glycerol test could be used to test whether enhanced eye velocity during vHIT testing is due to ELH, by selecting patients who showed enhanced eye velocity to horizontal head impulses and measuring their eye velocity before and at hourly intervals after oral intake of glycerol. We predicted that the glycerol should act to decrease the ELH and so to decrease the enhanced eye velocity. As a control we tested patients with enhanced eye velocity at hourly intervals before and after oral intake of comparable volumes of water.

## Materials and Methods

### Study Design and Participants

Patients were enrolled if they showed enhanced eye velocity on vHIT testing. The criterion level for enrolment was set as an initial VOR gain >1.29. That cutoff level was established by measuring the initial VOR gain in a group of healthy subjects where the mean value of initial VOR gain in 16 healthy asymptomatic subjects (32 ears) was 1.05 ± 0.118 (SD) (see Table 1 for demographic data on patients and healthy subjects). Thus, an initial VOR gain >1.29 is larger than 95% of the population [mean +2 standard deviations (25)], and so patients in whom the vHIT records for an impulse to one side showed an initial VOR gain >1.29 were enrolled in this study. We enrolled nine test patients (17 ears met criterion) and four control patients (6 ears met criterion). We stress that the inclusion criterion in this study was not based on diagnosis, but on this objective measure of enhanced eye velocity on vHIT testing. Table 1 shows the demographics of the patients and shows that the usual diagnosis was definite Menière's Disease based on Bárány Society guidelines (23). The diagnosis of Definite Menière's Disease according to the Bárány Society criteria requires (i) at least two episodes of spontaneous vertigo lasting between 20 min and 12 h, (ii) the presence of a low-frequency sensorineural hearing loss in the affected ear (at least 30 dB nHL in two neighboring frequencies <2 kHz) in the pure-tone audiogram before, during or after an attack, (iii) fluctuating auditory symptoms like fullness or tinnitus in the affected ear. In case the patient reports fluctuating hearing loss in the affected ear, but criterion (ii) is not fulfilled, the symptoms are classified as “probable Menière's Disease.” Glycerol test patients are G1–G9 and control patients are W1–W4. Two patients who met the inclusion criterion of an initial gain above 1.29 were diagnosed as having Vestibular Migraine according to the diagnostic criteria of the Bárány Society (24). A summary of the demographics of the group of 16 healthy subjects is also shown (Table 2).

**Table 1:** It shows the demographics of the patients and shows that the usual diagnosis was definite Menière's Disease based on Bárány Society guidelines (23).

**Patient no**.	**Gender**	**Age**	**Diagnosis**	**PTA**	**Ears included**^†^
**Glycerol**					
G1	Female	68	Bilateral definite MD*	78.5–50	L,R
G2	Female	54	Bilateral definite MD*	55–37.5	L,R
G3	Female	42	Vestibular migraine	12.5–10	L,R
G4	Female	43	Vestibular migraine	10–10	L,R
G5	Female	49	Right definite MD* – Left delayed endolymphatic hydrops	73.5–32.5	L,R
G6	Male	49	Right definite MD*	12.5–10	L,R
G7	Female	70	Bilateral definite MD*	35–62.5	L,R
G8	Female	31	Right definite MD*	36.25–10	R
G9	Male	35	Left probable MD	15–27.5	L,R
**Water**					
W1	Male	48	Right definite MD*	43.75–10	L
W2	Female	65	Bilateral definite MD*	21.25–22.5	L,R
W3	Female	32	Left definite MD*	10–32.5	L,R
W4	Female	44	Left probable MD	10–12.5	R

*Glycerol test patients are G1–G9 and control patients are W1–W4. Two patients who met the inclusion criterion of an initial gain above 1.29 were diagnosed as having Vestibular Migraine according to the diagnostic criteria of the Bárány Society (24)*.

“Ears included” refers to which sides showed enhanced eye velocity and were included in the statistical analysis. The asterisk * shows which patients had a diagnosis of definite Menière's Disease.

**Table 2:** It shows a summary of the demographics of the group of 16 healthy subjects.

**Subject no**.	**Gender**	**Age**
N1	Female	48
N2	Male	35
N3	Male	54
N4	Male	74
N5	Male	35
N6	Female	46
N7	Male	72
N8	Female	55
N9	Male	28
N10	Female	45
N11	Female	51
N12	Male	21
N13	Female	61
N14	Female	18
N15	Female	26
N16	Female	55
N17	Female	43

The patients were tested before and at hourly intervals after oral glycerol intake (test patients) or water intake (control patients). All the test patients were given orally 86% glycerol at a dosage of 1.5 mL/kg of body weight, dissolved 1:1 in physiological saline. The control patients received only water. Because vHIT testing is so fast and is not burdensome to patients, it was possible to test these patients at 4 successive epochs: before oral glycerol (or water) intake (time 0) and 1, 2, and 3 h after the intake. All patients were tested at quiescence.

### Ethics

All patients were informed about the procedure and the vestibular tests which were part of their clinical evaluation. The glycerol testing was carried out at the MSA ENT Academy Center in Cassino, and all procedures were performed in accordance with the Helsinki declaration, and were approved by the MSA ENT Academy Institutional Review Board, and all subjects and patients gave informed consent to the investigation and were free to terminate their participation at any time.

The glycerol experiment reported here yielded some results which were puzzling, so we reviewed earlier data from clinical vHIT testing of other patients tested during their routine clinical evaluation at Cassino or Donostia seeking examples of enhanced eye velocity in the responses of these patients. In each case the vHIT testing had been conducted with the patient's approval as part of the standard clinical assessment of their vestibular function. Since no novel or exceptional interventions were performed, simply the routine vHIT testing, only the approval of the local ethical committee for the corresponding institutions was required for the researchers.

### vHIT Testing

The function of the horizontal semicircular canals was measured using the horizontal video head impulse test (vHIT) (26) (OtosuiteV®, GN Otometrics, Denmark). Subjects were instructed to fixate an earth-fixed dot on the wall at 1 m distance in front of them. Room lighting conditions were adjusted to ensure that the pupil was small, and the pupil image was not affected by reflections at any point in the range of the head movement. At each testing epoch the clinician (LM) applied about 20 brief, rapid, horizontal head turns (head impulses) to each side, always starting from center, with unpredictable timing and direction with minimal bounce-back or overshoot at the end of the head impulse: that is each head impulse was “turn and stop.” The amplitude of the head rotation was about 10–15 deg, and the peak head velocity of the impulse was about 140–250 deg/s, with angular accelerations of between about 3,000 deg/s^2^ and 5,000 deg/s^2^. The sampling frequency was 250 frames/s. Video images were analyzed online to calculate eye position using a pupil detection method based on a center-of-gravity algorithm written in LabVIEW (National Instruments, Austin). Eye velocity and head velocity were recorded for each head turn. Eye velocity was obtained from a two-point differentiator and low-pass filtered (0- to 30-Hz bandwidth). Head accelerations were obtained using a Savitzky-Golay quadratic polynomial filter with a filter length of three points (corresponding to 12 ms) to smooth and differentiate the head-velocity data. The same process was used to obtain eye accelerations from the eye velocities. Because covert saccades rarely occur on the ascending phase of head velocity and because the initial VOR gain was based on a velocity point and not the usual area under the eye velocity curve, desaccading was not needed for the calculation of the initial VOR gain. At each testing epoch, the examiner sought to give head impulses with similar peak head velocities.

Three methods of calculating VOR gain from the slow phase eye velocity were used. (1) Area VOR gain: the area under the desaccaded eye velocity curve (27) divided by the area under the head velocity curve. This is the standard area VOR gain as used in the Otometrics Impulse system.

(2) The initial VOR gain defined as the peak eye velocity divided by the value of head velocity at the time of peak eye velocity. The onset of the head impulse was defined as 60 ms before the time of peak head acceleration. (3) the peak eye velocity divided by the head velocity at peak head acceleration, since this index may better reflect the effect of hydrops on cupula deflection.

**Figure 1:**
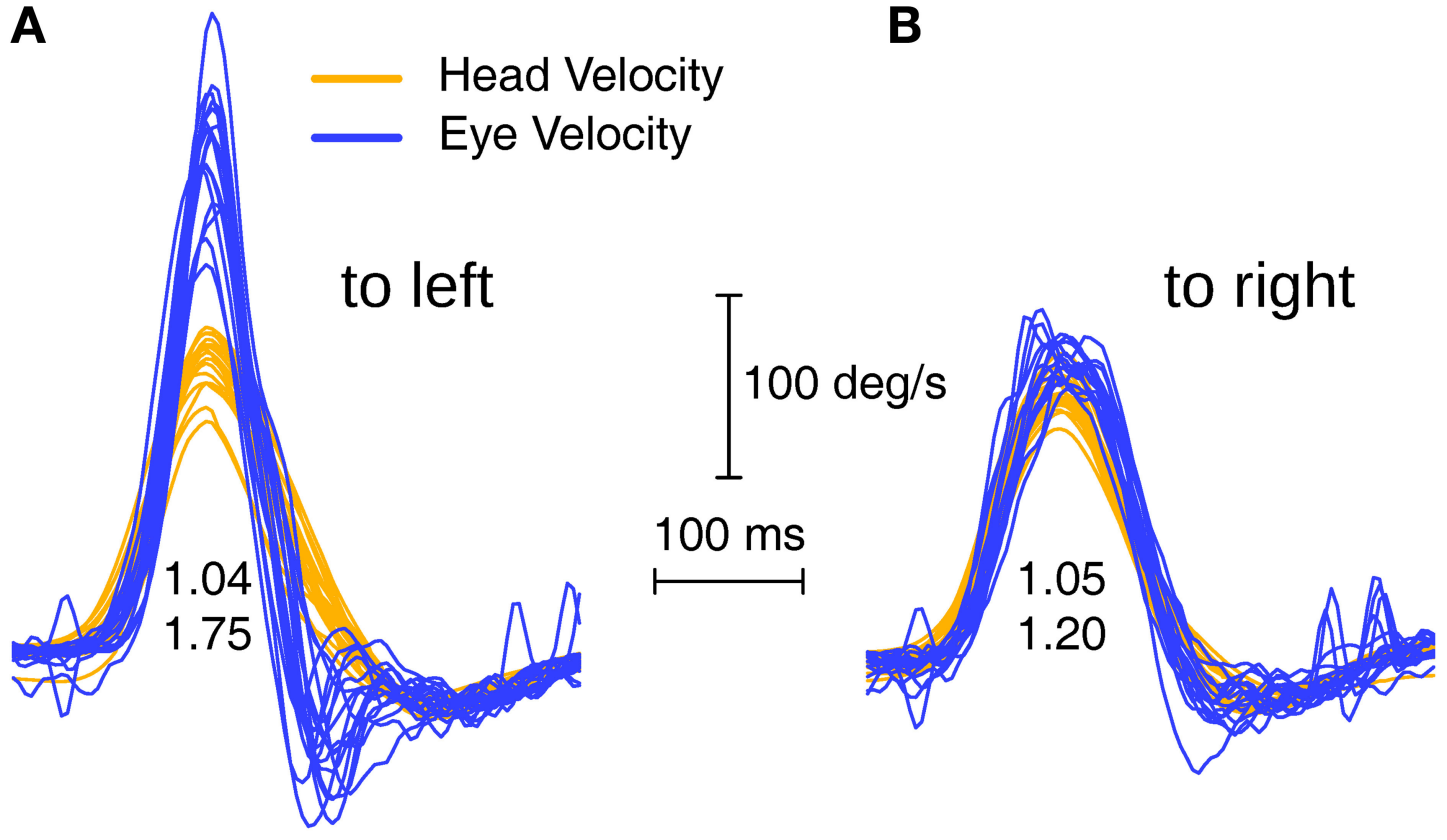
Superimposed eye velocity and head velocity records for head impulses to the left **(A)** and right **(B)** sides of a patient with enhanced eye velocity. In this example the usual indicator of VOR gain (area VOR gain) is 1.04 for the left side and 1.05 for the right side, which does not indicate the enhanced eye velocity which is so clear in the eye velocity time series on the left. Whilst the area VOR gains are similar for both L and R, the time series of eye velocities during the head impulses are completely different. In R the eye velocity tracks head velocity almost exactly, whereas in L there is an early enhanced eye velocity during the head acceleration followed by a steep decrease in eye velocity during impulse deceleration. To quantify the enhanced eye velocity on the left we calculated a parameter called initial VOR gain, which consists of the peak eye velocity divided by the value of head velocity at the time of peak eye velocity and the initial VOR gains here are 1.75 and 1.20.

In order to detect enhanced eye velocity, it is necessary to examine the time series records for individual trials–it can be concealed if only area VOR gain is examined because the eye velocity during the deceleration phase of the head impulse may cancel the enhanced eye velocity during the acceleration phase of the head impulse (see Figure 1). The enhanced eye velocity shown in individual trials occurs in the first part of the head impulse—from the onset to the peak head velocity (see Figure 1). To set an objective measure of what constitutes enhanced eye velocity we calculated the ratio of the peak eye velocity to the value of head velocity measured at the time of peak eye velocity. For this calculation, the peak eye velocity was defined as the highest eye velocity occurring in the time interval starting 40 ms before the time of peak head velocity, and ending 40 ms after the time of peak head velocity. We refer to this ratio of peak eye velocity to simultaneous head velocity as the initial VOR gain, and this was the objective measure of enhanced eye velocity used in this study.

As Figure 1 shows, area VOR gain does not faithfully reflect the enhanced eye velocity response of these patients, which is clear on inspection of the time series eye velocity records. For example Figure 1A shows a patient's vHIT responses on L where the actual trajectory of eye velocity during the head impulse is unlike the usual close match to head velocity of healthy subjects, and this patient recorded an area VOR gain of 1.04 but an initial VOR gain of 1.75, justifying our use of initial VOR gain as an objective indicator of enhanced eye velocity.

### Statistical Analysis

For each patient the initial VOR gain was calculated for every impulse, averaged across impulses at each testing epoch, and the averaged initial VOR gains across patients were analyzed with a one-way ANOVA with repeated measures using SPSS Version 26 (28). The data for the glycerol patients and water patients were analyzed separately. Shapiro-Wilk tests of normality (25) showed that the assumption of normality of distribution of the raw data was accepted in all conditions for both glycerol and water. Mauchly's test of sphericity (W) was not significant for both groups. The level of statistical significance was set at *p* < 0.05.

## Results

### Reduced Initial VOR Gain After Glycerol Intake

**Figure 2:**
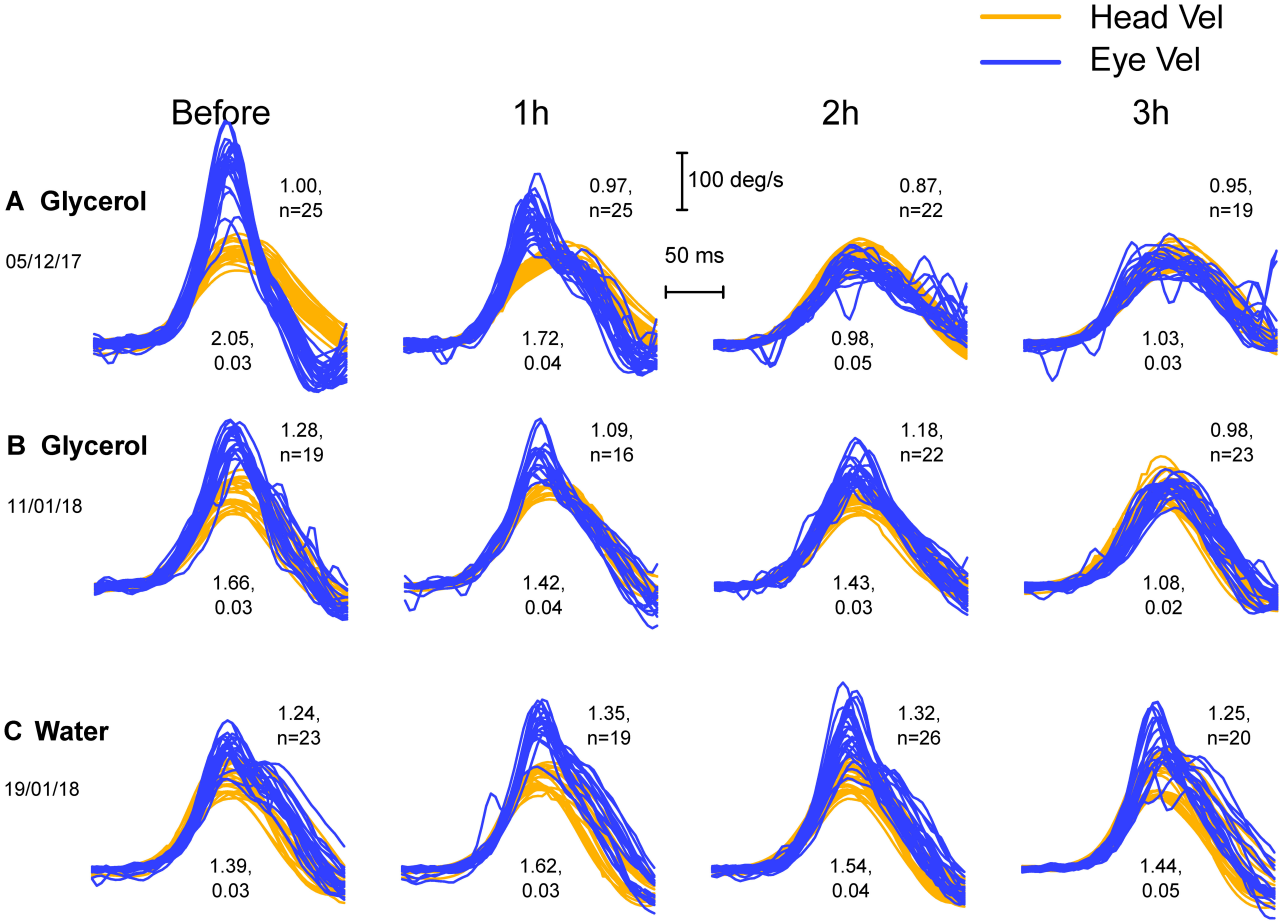
Three examples of the successive vHIT test results (plots which we term thumbnails) of two patients **(A,B)** with enhanced eye velocity before and at hourly intervals after oral glycerol intake (upper two rows) and for another patient **(C)** before and after water intake. Each thumbnail shows the superimposed records of eye velocity (dark blue) and head velocity (light orange) during the head impulse for many trials. There appear to be inflection points in the eye velocity records during the deceleration (explained below). Before glycerol (leftmost column) the three patients all have large initial VOR gains (values shown beneath the thumbnails), reflecting the enhanced eye velocity. The decrease in enhanced eye velocity in the glycerol patients after glycerol intake is clear, whereas there is no systematic decrease in the eye velocity or the initial VOR gain in the patient receiving water.

The responses of testing 17 sides (ears) of the nine patients whose data exceeded the criterion of initial VOR gain >1.29 were analyzed. Figure 2 shows examples of the raw data for three patients: the superimposed eye velocity and head velocity plots during standard head impulses of these patients before glycerol intake (upper two rows (A and B)) show enhanced eye velocity. Each separate image (which we term a thumbnail) shows the responses before and at hourly intervals after oral glycerol. For each set of impulses the mean initial VOR gain together with the standard error is shown beneath the impulses. The mean area VOR gain is shown to the right of the superimposed records. An example of the response of a control patient who received oral water instead of glycerol is shown in Figure 2C.

**Figure 3:**
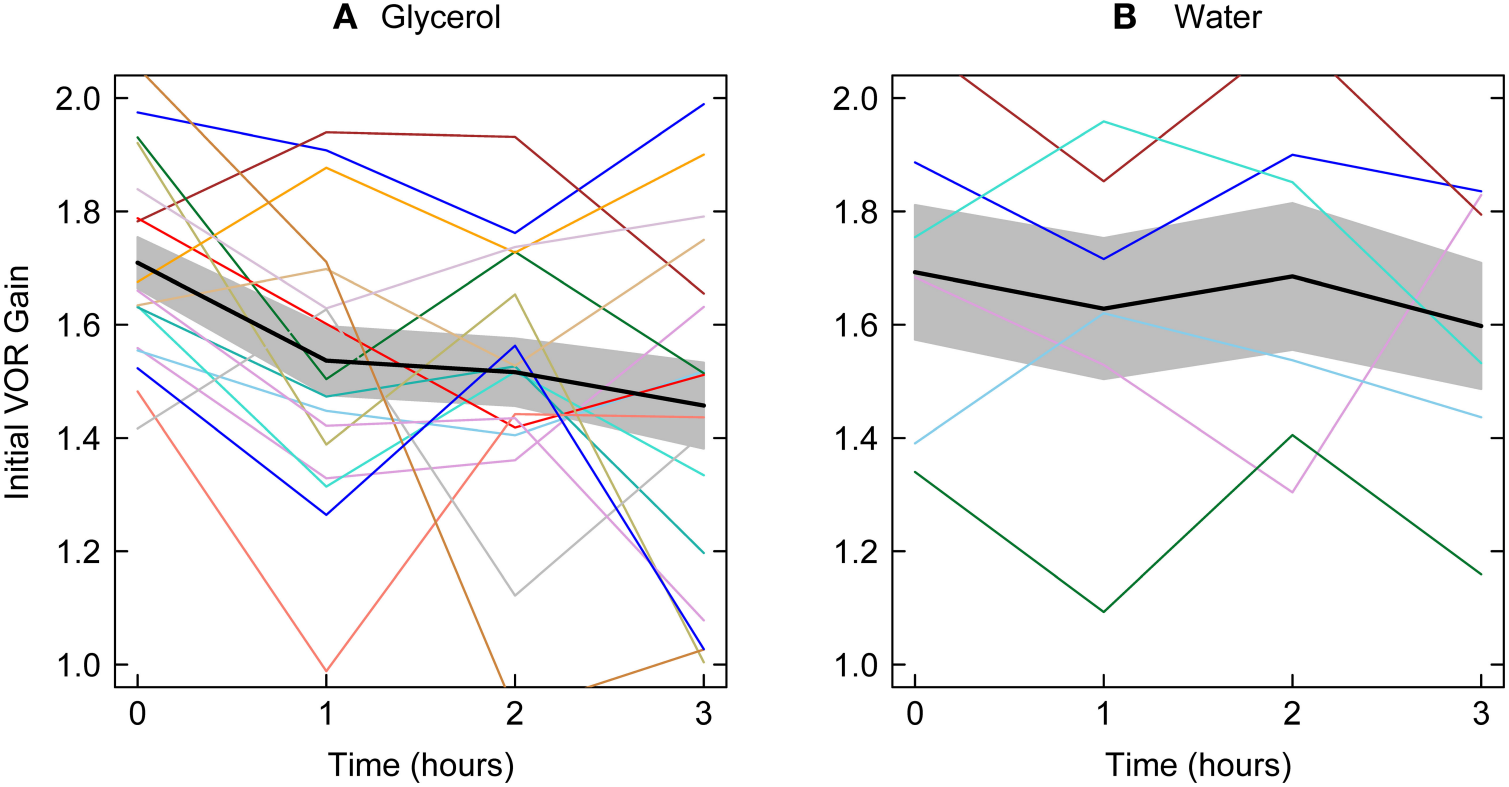
Initial VOR gain for all patients together with mean initial VOR gain across all patients tested at each testing epoch the gray bands show two tailed 95% confidence intervals for the means. Left panel—glycerol; right panel–water. The average decrease from before to 1 h after glycerol intake is significant and there is a significant linear decrease across the test epochs. There is no significant change in initial VOR gain for patients receiving water.

Figure 3 shows the average initial VOR gains at each testing epoch for patients receiving glycerol and those receiving water together with the means across the patients in the two groups (and two tailed 95% confidence intervals – gray bands). The ANOVA on the 17 glycerol ears was significant: *F* = 4.72, *p* = 0.006 with the contrast for linear trend showing that for the glycerol data there was a significant linear decrease in initial VOR gain across the testing intervals *F* = 8.04, *p* = 0.012. A total of 13/17 ears tested showed a decrease in VOR 1 h after glycerol. The ANOVA for the water control was not significant: *F* = 0.454, *p* = 0.718). Only 17 ears of nine patients were measured since the last ear did not meet the inclusion criterion of an initial VOR gain above 1.29.

Using the VOR gain specified by peak eye velocity divided by the head velocity at time of peak head acceleration also showed that glycerol caused a significant decrease in VOR gain.

### Absence of Corrective Saccades After the Enhanced Eye Velocity

**Figure 4:**
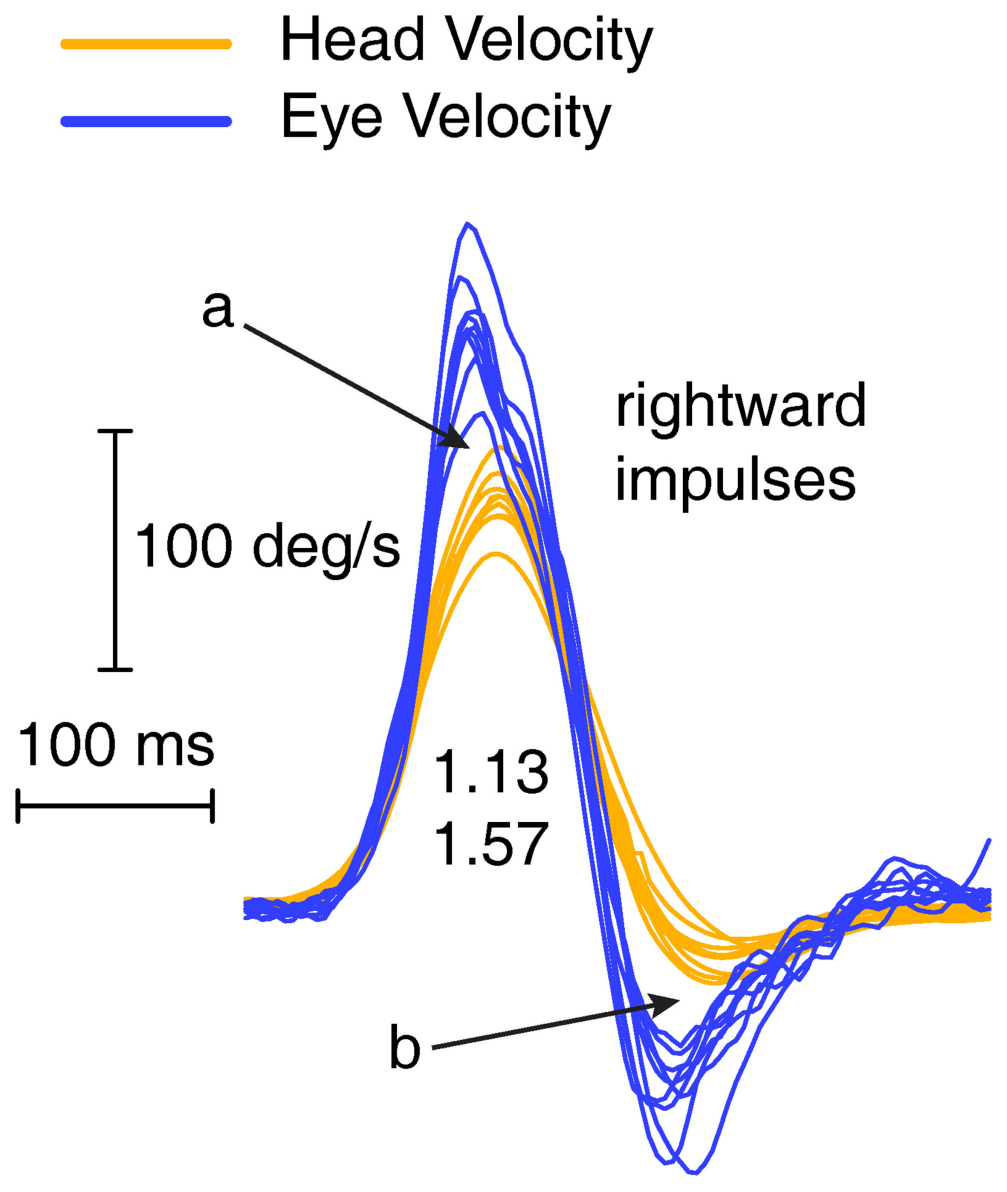
Enhanced eye velocity (a) during acceleration may be canceled by decreased eye velocity during deceleration (b) leading to a final eye position which stays close to the target.

If eye velocity closely matches head velocity during a head impulse, then at the end of the impulse the eye will be on target, so there will be no gaze error and so no corrective saccade will be necessary. The Otometrics system (ICS Impulse) shows area gain, which is the ratio of the change in eye position to the change in head position. The change in head position is the integral of head velocity during the head impulse—i.e., the area under the head velocity curve. The change in head position is matched and corrected for by the change in eye position, so at the end of the impulse the eye is on the target and no saccade is necessary. However, the area gain for the entire head impulses may be close to 1, although there may be a clearly enhanced eye velocity at the start of the impulse, then in the second half of the impulse the eye velocity is regularly less than the decreasing head velocity (see Figure 4), thus acting to cancel out the position error caused by the enhanced eye velocity on the acceleration phase. The result is that the area between eye velocity and head velocity (arrow a in Figure 4) is about the same as the (opposite) area between eye velocity and head velocity in the decelerating phase (arrow b), so these two areas effectively cancel and the eye position at the end of the head impulse will be on target, so no corrective saccade will be necessary even though the trajectory of the eye velocity during the head impulse is so different from the usual response (see also Figure 1A). While the graphical records may show this, the area gain value may not. These examples show why we derived and used this gain measure—initial VOR gain—which reflects the enhanced eye velocity during the initial part of the head impulse.

### Saccade-Like Responses

**Figure 5:**
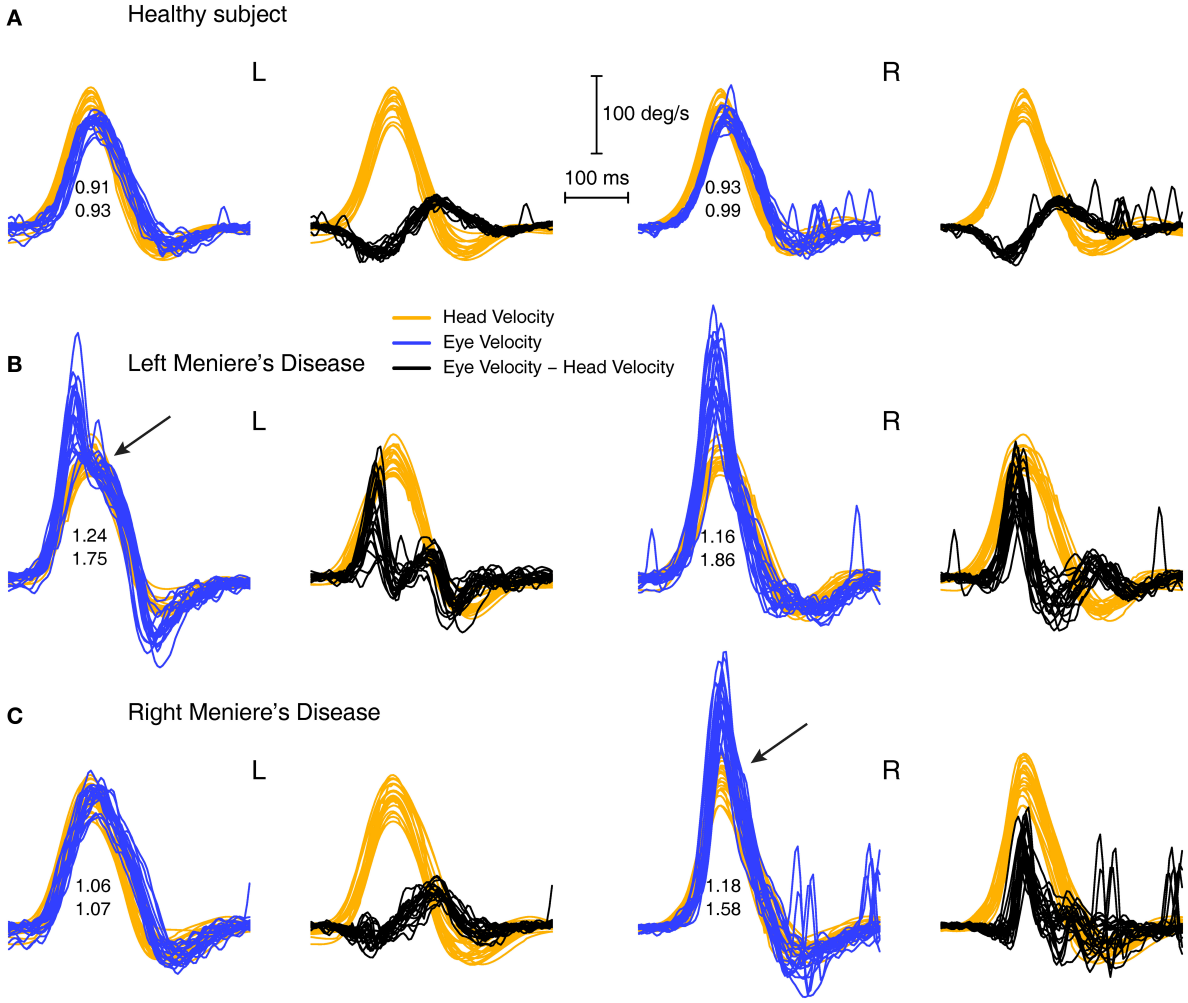
Overlaid traces of eye velocity (blue) and head velocity (orange) and the difference between eye and head velocity (black) for head-impulse data for two patients with high VOR gain **(B,C)** and for a healthy subject **(A)**. Responses for the patients shows a pronounced peak in the difference between eye and head velocity (black traces) and we term this the “saccade-like response.” In healthy subjects this difference stays close to zero or is even negative. Both sets of enhanced eye velocity for the patients show an inflection point in the eye velocity time series (shown by arrows) which would suggest the end of a saccade-like response whose point of initiation is hidden in the very rapidly increasing eye velocity during head acceleration.

In some patients the enhancement of eye velocity during the head impulse stimulus is fairly uniform at all head velocities (see Figure 4). However, the records from other patients show what appears to be a saccade-like response which is added to the eye velocity during the initial head acceleration. It is a compensatory saccade and not an anticompensatory quick phase since the saccade direction is opposite to the direction of head turn. (A quick phase of nystagmus is a rapid eye movement in the same direction as head turn and so is anticompensatory). This response appears to be a very early covert saccade. Such a saccade-like response is very difficult to detect during the increasing head velocity at the start of the head impulse because the saccadic velocity is very close to the high eye velocity at the start of the head impulse. However, an inflection at the end of the saccadic-like responses (arrows in Figure 5) is a tell-tale sign of the addition of this saccade-like response during the head impulse. These two response modes suggest there may be two mechanisms operating to produce VOR gain enhancement. The first being the enhancement shown by fluid dynamic modeling (5), the second being a mechanism which generates this saccade-like response. We sought to try to clarify further this saccade-like mechanism and consider what may cause it.

By plotting the time series of the difference between eye velocity and head velocity during the whole head impulse (Figure 5, second and fourth columns) for a healthy subject (A) and patients (B and C), this saccade-like response is clearly shown in the patient records (B and C). The difference between eye velocity and head velocity effectively cancels the slow compensatory eye velocity response and leaves the saccade-like response exposed. For horizontal head turns for healthy subjects (top row), the typical records have the eye velocity matching head velocity quite closely, so the difference (E–H) curves are flat or even concave (probably because of the small effect of latency of the eye velocity response). These early saccade-like responses occur rarely in healthy subjects (Figure 5A) but are found and are highly repeatable in patients (e.g., arrows in Figure 5 above).

**Figure 6:**
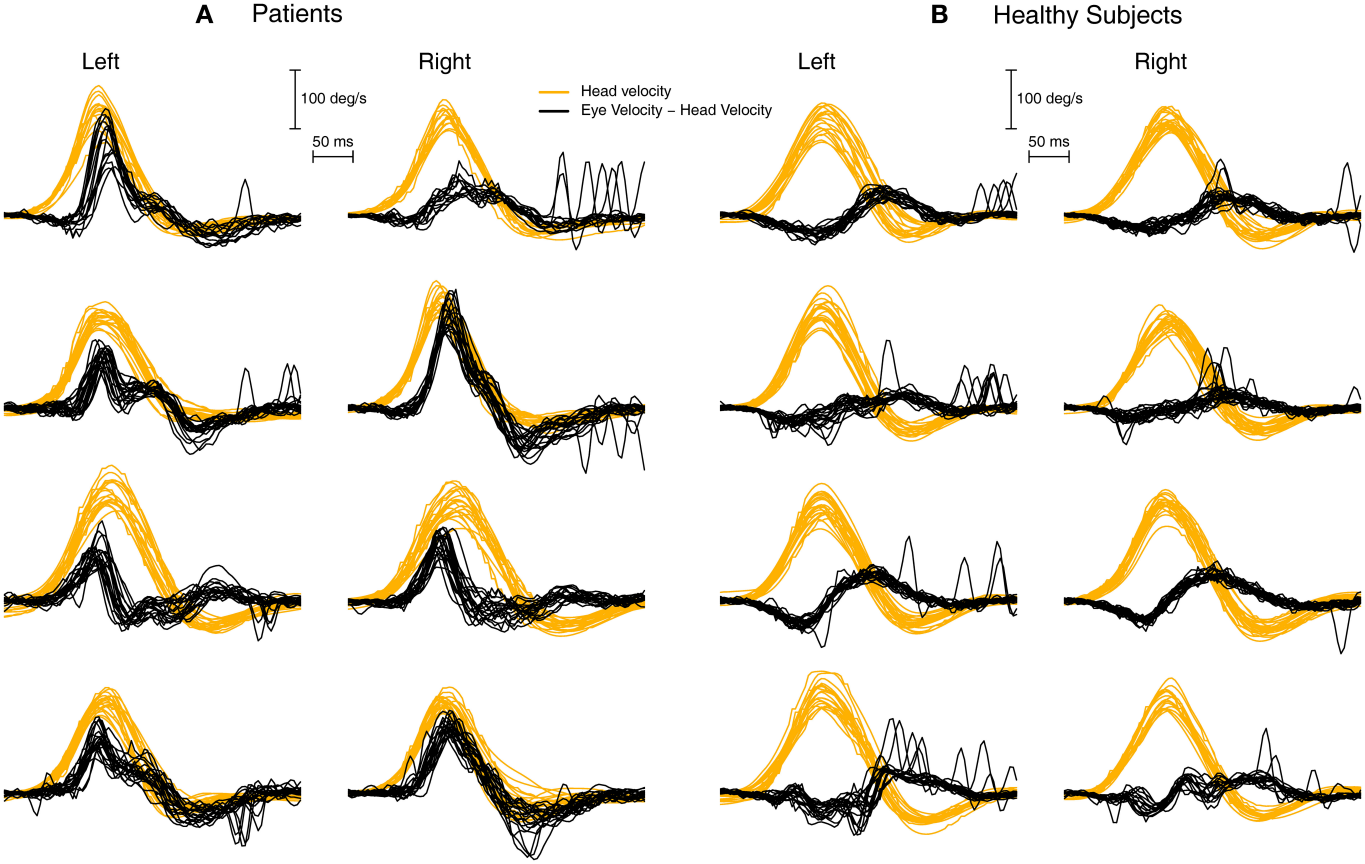
To show the value of plotting the difference between eye velocity and head velocity for patients **(A)** and healthy subjects **(B)**. Each row shows the E-H plot for one patient and one healthy subject. The very different E-H plots between the groups is clear.

Figure 6 shows several examples of the raw data and the corresponding plot of the difference between eye velocity and head velocity for four patients and four healthy subjects to establish the consistency of the response patterns and the usefulness of this mode of plotting the responses of head impulse testing.

## Discussion

These results show that there is an enhanced eye velocity during head impulse testing and that oral glycerol on average results in a significant decrease in this enhanced eye velocity. Consuming a comparable volume of water does not result in a significant decrease of enhanced eye velocity. Other evidence shows that glycerol acts to reduce ELH, so our result implies that enhanced eye velocity on vHIT testing may be an indicator of endolymphatic hydrops. In the following we consider major questions about these results—the evidence that enhanced eye velocity occurs, whether it is artifactual, the variability of patient responses, what could trigger the enhancement.

### Patient Groups

The enhanced eye velocity is not restricted to patients with MD but occurs in other patient groups: some patients diagnosed as having vestibular migraine show enhanced eye velocity. This is in accord with the hydrops model put forward above because ELH occurs not only in MD but can also occur in other conditions (29, 30). The group which is poorly represented in our testing is healthy subjects because enhanced eye velocity was so rarely found in their results, as has been reported in a large study (7).

Why are there such different patterns of enhanced eye velocity responses during head impulses not seen by all clinics? We consider that patient selection is an important factor. Some clinics only see patients who are well-advanced in the disease (31, 32). Other clinics test patients who are at the very earliest stage of inner ear disease (33), and it may be that the enhanced eye velocity is more salient early in the disease. Up to now VOR gains >1 tend to have been discounted as measurement error in VHIT records but see Figure 4 of Carey et al. (6) which shows enhanced eye velocity recorded with scleral search coils so there was no goggle slippage.

### Artifacts

Artifacts can occur during head impulse testing (8, 34), for example calibration errors or goggle slippage. The enhanced eye velocity we report here is not due to calibration errors or goggle slippage. There are two main reasons: the operator was very well-trained in carrying out the head impulse test and checked calibration and the tightness of the goggles whenever enhanced eye velocity occurred. Can loose straps cause a similar enhanced eye velocity? No. We have systematically loosened and tightened goggle straps without being able to duplicate the highly reliable enhanced eye velocity response pattern we have shown above.

The patient responses themselves show the enhancement is not artifactual because these responses differentially occurred in patients and not in healthy subjects. Furthermore, if it occurred in a patient it was highly likely to reappear when that patient was retested [see also Rey-Martinez et al. (4)]. Furthermore, if it were simply artifactual then it is not clear why the enhancement should systematically decrease after oral glycerol intake but not water intake.

### Variability

In some patients with enhanced eye velocity there are only minimal changes in eye velocity after oral glycerol in this vestibular version of the glycerol dehydration test, just as some patients only show minimal changes in the auditory version of the glycerol dehydration test for ELH (35). The variability of these results is not surprising given the variability of the hydrops of the membranous labyrinth now being revealed by MRI scans (36). As is becoming clear from imaging of labyrinths with ELH, the hydrops can vary greatly in the location of greatest swelling from one individual to another—in some patients the hydrops is mainly around the utricle, in others it may be mainly around the cochlea (36).

Clearly differential enlargement of the membranous labyrinth will have very different effects on the way in which the enlarged fluid volume can affect the canal response. So we would expect that some patients with enlarged utricular volume would show large effects of glycerol on the enhanced eye velocity, whereas others with predominantly cochlear hydrops would show little effect. We do not yet have sufficiently precise estimates of the exact location of the enlarged membranous labyrinth volume to make predictions relating the hydrops to enhanced eye velocity, but this is a prediction for future research. Also, the exact effect of glycerol on the likely non-uniformly enlarged membranous labyrinth is unknown—glycerol may differentially affect different structures. The auditory glycerol dehydration test uses threshold measurement, and it has been suggested that some of the variability in that test is due to patient expectancy effects (35). Glycerol affects the objective physiological index of ELH, the ECochG, but there is still considerable variability between patients (17).

### The Saccade-Like Response During Head Acceleration

In routine vHIT testing compensatory covert saccades may occur after the end of the impulse (overt saccades) or during the decreasing eye velocity of a head impulse (covert saccades).

In both cases saccades are error correcting eye movements to return the patient's gaze position to the target. Compensatory covert saccades are relatively easy to identify visually (and computationally) because the saccade direction and velocity is opposite in direction to the decreasing slow phase eye velocity in the “deceleration” phase of the head impulse, so the inflection at the start of the covert saccade is apparent (37). However, a compensatory covert saccade during the increasing eye velocity on the acceleration phase of the head impulse is difficult to detect since the saccade velocity is in the same direction as the compensatory (“slow phase”) eye velocity. In that case a covert saccade is just an increment on the increasing eye velocity and so difficult to detect, although our technique of plotting the difference between eye velocity and head velocity shows this saccade-like response clearly.

The important questions are what could trigger such a consistent response pattern? These are not just anticipatory saccades since they are invariably in the correct compensatory direction, even though the direction of successive head impulses was randomized. What could trigger this response since their latency is so short? The angular jerk at the onset of the head impulse could act as a trigger. If canal receptors have enhanced sensitivity, e.g., are jerk sensitive, they could trigger such an early rapid saccade.

In our data the patients had enhanced eye velocity during the initial part of the head impulse, and one possibility is that this is due to the fact that the transient semicircular canal receptor and afferent system (38) is sensitized. The evidence for this is shown by the comparison of initial VOR gain in normals vs. initial VOR gain in patients. So, one hypothesis is that the earliest part of the response in vHIT is due to the transient canal system—the irregular afferents synapsing on type I receptors at the crest of the crista (39–41). So, loss of these receptors—for example after systemic gentamicin which preferentially attacks type I receptors (42–44)—should lead to a poor initial response. That has been shown in patients who have received gentamicin either systemically (45) or intratympanically (6). Conversely, if those receptors were to be sensitized there should be a larger initial gain. Is there evidence for such sensitization? There is indirect evidence that these transient receptors/afferents may be sensitized in MD patients (46) who showed enhanced eye velocity response to abrupt onset galvanic stimulation (47, 48).

Another stimulus which could act as a trigger for enhanced eye velocity and the saccade-like response is tangential linear acceleration (or tangential jerk) acting on otolithic receptors.

Tangential linear acceleration during an angular rotation is defined as the product of the angular acceleration x the radius (from the axis of the rotation to the otolithic receptors), and in usual turntable testing this is insignificant because R is so small (3.76 cm) (49) and the angular acceleration is also small (perhaps 100 deg/s^2^). However, with head impulse testing, the tangential linear acceleration is large because the angular acceleration is of the order of 3,000 deg/s^2^. The onset of that linear acceleration or linear jerk could serve as a trigger. Tangential linear acceleration is not usually considered in animal physiological studies recording otolithic responses because the radius of animal heads is even smaller than for human heads and large angular accelerations are not usually given.

## Conclusion

Some patients show consistent enhanced eye velocity responses on vHIT testing, and this study indicates that such enhanced eye velocity may be an indicator of endolymphatic hydrops. Oral intake of glycerol which acts to constrict the membranous labyrinth enlargement also reduces the enhanced eye velocity. The reduction of the enhanced eye velocity by glycerol serves to confirm the indication of hydrops. We have presented a measure of enhanced eye velocity—initial VOR gain–and a new technique for identifying saccade-like responses in the individual trials.

## Data Availability Statement

The raw data supporting the conclusions of this article will be made available by the authors, without undue reservation.

## Ethics Statement

The studies involving human participants were reviewed and approved by MSA ENT Academy Institutional Review Board. The patients/participants provided their written informed consent to participate in this study.

## Author Contributions

IC conceived the study and wrote the paper. AB and IC conducted the statistical analysis. LM carried out the testing. LM, JR-M, and JD made contributions to the interpretation. All authors contributed to the manuscript and read and approved the submitted version.

## Conflict of Interest

The authors declare that the research was conducted in the absence of any commercial or financial relationships that could be construed as a potential conflict of interest.

## Abbreviations

ECochG: electrocochleography
ELH: endolymphatic hydrops
VOR: vestibulo-ocular reflex
HIT: head impulse test
vHIT: video head impulse test.
